# Materia-Medica for the Use of Students

**Published:** 1874-11

**Authors:** 


### Books Reviewed.

*Materia Medica for the use of Students.* By JOHN B. BIDDLE, M. D., etc. Sixth Edition Revised and enlarged, with Illustrations. Philadelphia: Lindsay & Blakiston, 1874.

The standard Materia Medica with a large number of medical students in the United States is Biddle's. But a little more than a year has elapsed since the publication of the fifth edition, and yet the author finds that the edition has been exhausted and that a new one is called for.

He has found it necessary to re-write some portions of the work, more especially those comprising therapeutics, and has made important changes in others. Transfusion is spoken of, and Aveling's Apparatus is figured. Some new articles of the Materia Medica have been added, and in its present revised condition the work will be found to be a valuable and convenient text book of Materia Medica.

